# Immunohistochemical comparison of lateral bone augmentation using a synthetic TiO_2_ block or a xenogeneic graft in chronic alveolar defects

**DOI:** 10.1111/cid.13143

**Published:** 2022-10-12

**Authors:** Minh Khai Le Thieu, Sabine Stoetzel, Maryam Rahmati, Thaqif El Khassawna, Anders Verket, Javier Sanz‐Esporrin, Mariano Sanz, Jan Eirik Ellingsen, Håvard Jostein Haugen

**Affiliations:** ^1^ Department of Periodontology, Institute of Clinical Dentistry, Faculty of Dentistry University of Oslo Oslo Norway; ^2^ Department of Biomaterials, Institute of Clinical Dentistry, Faculty of Dentistry University of Oslo Oslo Norway; ^3^ Department of Experimental Trauma Surgery, Faculty of Medicine Justus‐Liebig University Giessen Giessen Germany; ^4^ Periodontology University Complutense of Madrid Madrid Spain; ^5^ Department of Prosthetics and Oral Function University of Oslo Oslo Norway

**Keywords:** animal experimentation, bone regeneration, bone substitutes, guided tissue regeneration, immunohistochemistry, xenografts

## Abstract

**Objectives:**

To evaluate osteogenic markers and alveolar ridge profile changes in guided bone regeneration (GBR) of chronic noncontained bone defects using a nonresorbable TiO_2_ block.

**Materials and Methods:**

Three buccal bone defects were created in each hemimandible of eight beagle dogs and allowed to heal for 8 weeks before GBR. Treatment was assigned by block randomization: TiO_2_ block: TiO_2_‐scaffold and a collagen membrane, DBBM particulates: Deproteinized bovine bone mineral (DBBM) and a collagen membrane, Empty control: Only collagen membrane. Bone regeneration was assessed on two different healing timepoints: early (4 weeks) and late healing (12 weeks) using several immunohistochemistry markers including alpha‐smooth muscle actin (α‐SMA), osteopontin, osteocalcin, tartrate‐resistant acid phosphatase, and collagen type I. Histomorphometry was performed on Movat Pentachrome‐stained and Von Kossa/Van Gieson‐stained sections. Stereolithographic (STL) models were used to compare alveolar profile changes.

**Results:**

The percentage of α‐SMA and osteopontin increased in TiO_2_ group after 12 weeks of healing at the bone‐scaffold interface, while collagen type I increased in the empty control group. In the defect area, α‐SMA decreased in the empty control group, while collagen type I increased in the DBBM group. All groups maintained alveolar profile from 4 to 12 weeks, but TiO_2_ group demonstrated the widest soft tissue contour profile.

**Conclusions:**

The present findings suggested contact osteogenesis when GBR is performed with a TiO_2_ block or DBBM particulates. The increase in osteopontin indicated a potential for bone formation beyond 12 weeks. The alveolar profile data indicated a sustained lateral increase in lateral bone augmentation using a TiO_2_ block and a collagen membrane, as compared with DBBM and a collagen membrane or a collagen membrane alone.

**What is known**

Lateral bone augmentation in chronic alveolar defects using a bone graft material usually leads to contact osteogenesis. Histological analysis may be used to describe the morphological situation, but gives limited information of the potential for bone formation.

**What this study adds**

Immunohistological data obtained by microtome sectioning MMA embedded samples indicates potential for further bone formation beyond 12 weeks healing.

## 1 INTRODUCTION

Guided bone regeneration (GBR) employs a membrane as a mechanical barrier to avoid soft tissue involvement in the healing process. Thereby, the osteogenic potential that achieves bone augmentation arises from the bony defect's walls. The standard protocol commonly combines graft material with a membrane to create space for new bone formation and avoid soft tissue infiltration. Although some clinical studies have shown predictable bone gain,^1^, ^2^ others have reported less bone formation when using a graft material compared with when using the membrane alone.^3^, ^4^, ^5^, ^6^

In a previous in vivo experimental study,^7^ GBR with a collagen membrane alone was compared with deproteinized bovine bone mineral (DBBM) and a ceramic TiO_2_ scaffold. Less bone formation was observed in the TiO_2_ and DBBM groups when compared with membrane alone group at the final follow‐up time point after 12 weeks of healing. However, the groups using bone replacement grafts demonstrated increased volumetric lateral bone augmentation. In this study, however, these findings were assessed by microcomputed tomography and histomorphometry, where information could not be obtained on the osteogenic dynamics during this observation period.

Osteogenesis during GBR undergoes a complex process of fine‐tuned coordinated phases. Initially, an inflammatory phase occurs where leukocytes, including macrophages, are recruited. Subsequently, new blood vessels form and osteoblasts deposit an extracellular matrix. A matrix maturation phase then follows before the final mineralization phase, where osteoblasts remodel woven bone into mature lamellar bone.^8^, ^9^ The different stages of osteoblast growth and differentiation can be identified either by specific gene expression or by quantifying protein secretion using histochemical methods. In the proliferation phase, there is a characteristic peak in collagen type 1 during the formation of bone extracellular matrix. Subsequently, during the matrix maturation phase, collagen type I decreases and osteopontin and osteocalcin increase, reaching their maximal expression during the mineralization phase.^10^ Other histochemical markers like tartrate‐resistant acid phosphatase (TRAP) and alpha‐smooth muscle actin (α‐SMA) represent biological cues for osteoclast activity and blood vessel formation, respectively. Hence, the quantification of these markers by histochemical analysis can study the stages of bone development, including the required neoangiogenesis and bone remodeling processes.

Therefore, the primary aim of this study was to assess the osteogenic potential by evaluation and quantification of osteogenic markers by immunohistochemistry (IHC), when using a bioabsorbable membrane alone compared with the use of either an additional TiO_2_ block or DBBM particulates as bone replacement grafts. The secondary aim was to assess the changes in the alveolar ridge soft tissue profile from baseline to 4 weeks and 12 weeks by histomorphometry.

## 2 MATERIALS AND METHODS

### 2.1 Materials

**FIGURE 1:**
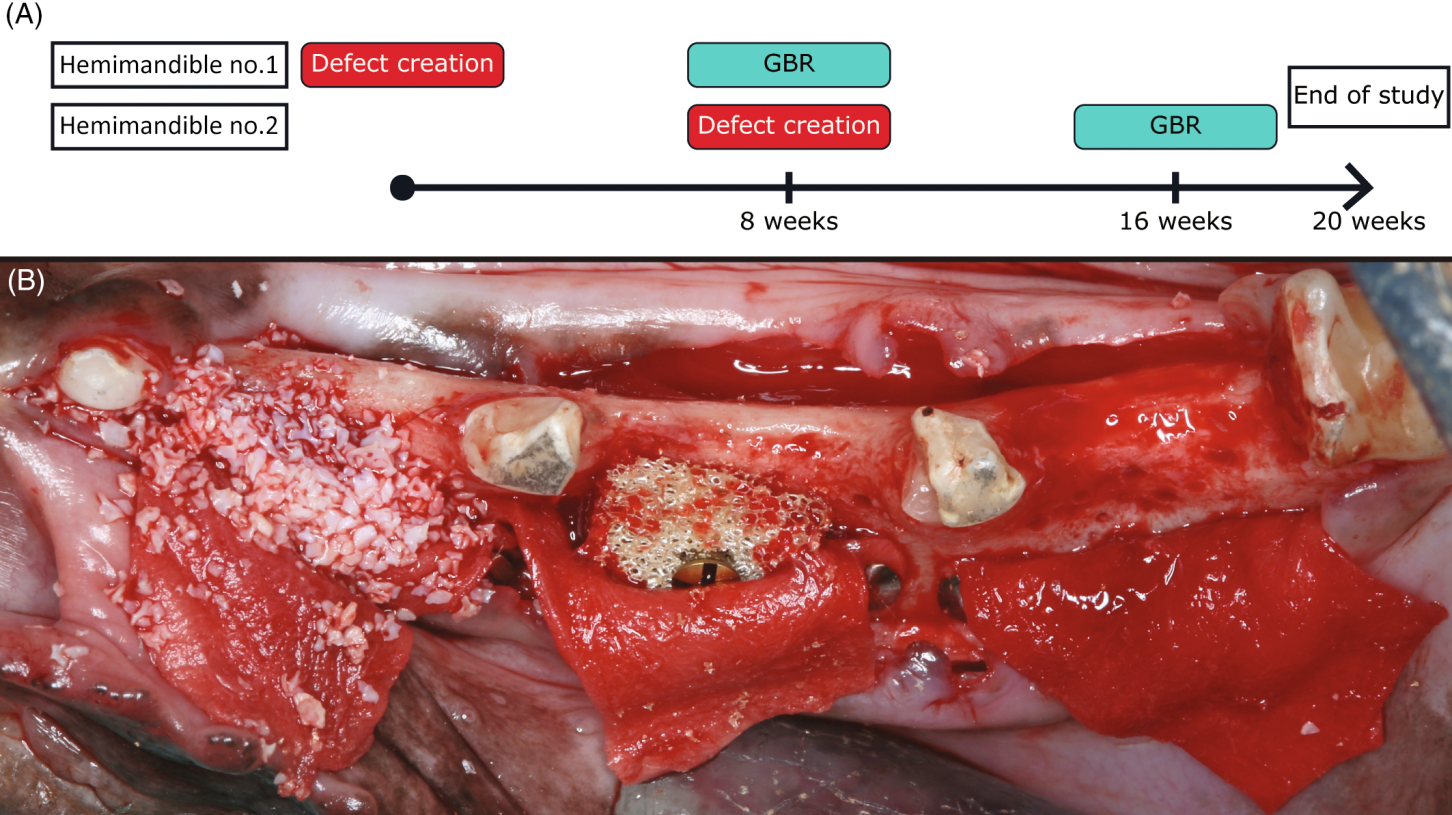
(A) Timeline of the study design. (B) Guided bone regeneration (GBR) procedure on noncontained defects. Showing the anterior defect with deproteinized bovine bone mineral particulates, middle defect with a TiO_2_ block secured with a fixation screw and the empty posterior defect. Cortical perforations were performed at all recipient sites. All sites were covered with a collagen membrane stabilized by pin fixation.

A timeline of the study design is shown in Figure 1A. Porous ceramic TiO_2_ scaffold blocks were produced by foam replication. Teeth extraction and defect creation were performed according to the protocol of Sanz and colleagues^11^ Three standardized defects were created in each hemi mandible to a standardized box shape measuring 10 mm mesiodistally, 10 mm apicocoronally, and 5 mm buccolingually using bone burs under copious saline irrigation, and left to heal for 8 weeks prior to GBR procedures. After 8 weeks healing, GBR surgery was performed on the cortical bone of the one‐wall defects. All recipient sites were perforated with round burs, treated with GBR materials (Figure 1B) and primary healing was obtained. The hemimandibles were allocated to either 4 or 12 weeks of healing time. The three defect sites were randomly allocated to the following treatment groups:

1. TiO_2_ block: TiO_2_ scaffold (Corticalis AS, Oslo, Norway) covered by a collagen membrane (BioGide®; Geistlich Pharma AG, 6110 Wolhusen, Switzerland)
2. DBBM particulates: A xenograft of DBBM (Bio‐Oss® Granules 0.25–1 mm, Geistlich Pharma AG, 6110 Wolhusen, Switzerland) covered by a collagen membrane (BioGide®; Geistlich Pharma AG, 6110 Wolhusen, Switzerland)
3. Empty control: A collagen membrane covered the defect (BioGide®; Geistlich Pharma AG, 6110 Wolhusen, Switzerland)

The collagen membranes were fixated at the apical edges by titanium pins (Botiss titan pins 3 mm, Straumann AG, CH‐4002 Basel, Switzerland) and the TiO_2_ blocks were fixated with a bone block fixation screw 1.5 mm in diameter and 10.0 mm in length (Straumann AG, CH‐4002 Basel, Switzerland), adapted to fit the alveolar width. Further details regarding the study design and material preparation have been described previously.^7^

### 2.2 Animal handling

This experimental in vivo investigation was designed in accordance with the ARRIVE (Animal Research: Reporting of In Vivo Experiments) guidelines for preclinical research.^12^ Its protocol was approved by the Ethical committee at the Jesús Usón Minimally Invasive Surgery Centre (Caceres, Spain) and by the Director General of Agriculture and Livestock (approval code: 2018209020003431).

A total of eight female beagle dogs (weight, 11–14 kg) were used. The animals were kept in a 12:12‐light/dark cycle at 21–22°C in individual kennels and fed on soft pellet diet, being daily monitored by a veterinarian. Prior to the study, all animals were inspected to ensure the absence of oral disease or any dental conditions that would preclude bone regenerative intervention. Two weeks prior and throughout the study, the animals were monitored for any signs or symptoms of systemic disease.

During the surgical interventions, the animals were premedicated with acepromazine (0.05 mg/kg/i.m., Calmo Meosan, Pfizer, Madrid, Spain) and morphine (0.3 mg/kg/i.m., Morfina Braun 2%, B. Braun Medical, Barcelona, Spain). Then they were first sedated with propofol (2 mg/kg/i.v., Propovet, Abbott Laboratories, Kent, UK) and then general anesthesia was applied using 2.5%–4% of isoflurane (Isoba‐vet, Schering‐Plow, Madrid, Spain) under mechanically induced respiration. The animals were then infiltrated locally with Lidocaine 2% with epinephrine 1:100 000 (2% Xylocaine Dental, Dentsply, York, Pennsylvania) as local anesthetic. After surgeries, the animals were administered Morphine (0.3 mg/kg/i.m.) for the first 24 h and meloxicam (0.1 mg/kg/s.i.d./p.o., Metacam, Boehringer Ingelheim España, Barcelona, Spain) for 3 days after surgeries to control pain. Antibiotic therapy with amoxicillin (22 mg/kg/s.i.d./s.c., Amoxoil retard, Syva, León, Spain) was used for 7 days after the surgeries.

At the allocated healing times the animals were euthanized with a lethal dose of sodium pentobarbital (40–60 mg/kg/i.v., Dolethal, Vetoquinol, France) and their mandibles were dissected and fixed in formalin.

### 2.3 Histological preparation and histomorphometric analysis

Samples were dehydrated in an ascending series of alcohol and xylene baths before embedding in methyl methacrylate and polymerizing at −20°C. The resulting embedded defect sites were divided into two halves. One was allocated for microcomputed tomography and undecalcified histomorphometry, and was utilized for the recently published results.^7^ The other half was allocated to microtome sectioning and staining with Movat Pentachrome and Von Kossa/Van Gieson. At least four representative sites per treatment group per time point were included for IHC staining. These samples were sectioned in the middle of the defect in buccolingual direction in 5‐μm thickness onto Kawamoto's film (SECTION‐LAB Co. Ltd., Hiroshima, Japan) using a motorized rotary microtome (Thermo/Microm HM 355 S, Thermo Scientific GmbH, Karlsruhe, Germany).

Movat Pentachrome stain was used to quantify collagen.^13^ Von Kossa/Van Gieson staining was used to quantify the extracellular matrix mineralization.^14^

Histomorphometry was performed on slides scanned using an AxioScan Z1 (Carl Zeiss, Germany) and analyzed using ImageJ (ImageJ 1.53f51, National Institutes of Health, USA). The Trainable Weka Segmentation plugin for ImageJ was used to quantify the stained areas, as described by Malhan and colleagues^15^ In these slides, three regions of interest (ROI) were chosen. (1) The buccal half of the alveolar bone, including the grafted area, measured from the tip of the alveolar crest and extending 10 mm apically (ROItot). (2) An area representing the interface between bone and the graft expanded 200 μm in both buccal and lingual directions (ROI400 μm). In the empty control group, the area between bone and soft tissue was measured, and graft materials were excluded if present. (3) An area was determined from the same interface as for the ROI400 μm but only expanded 20 μm in both buccal and lingual directions (ROI 40 μm).

### 2.4 Enzyme histochemical and immunohistochemical preparation and analysis

Sections were deplastified prior to staining. To show TRAP activity, sections were incubated in Sodium Acetate buffer, Naphthol‐AS‐TR phosphate (N6125‐1G, Sigma, Germany) and Sodium Tartrate (Merck, Germany) at 37°C for 60 min.

IHC was done using the following primary antibodies (Abcam Company, Cambridge, UK): rabbit monoclonal (EPR53) to α‐SMA, rabbit polyclonal (OAA100188) to osteopontin, mouse monoclonal (LS‐C83497‐100) to osteocalcin and rabbit monoclonal (EPR7785) to collagen type I.

To study the blood vessel formation, α‐SMA, osteopontin, osteocalcin, and collagen type‐I were diluted in DAKO‐Diluent (S 0809), 1:400, 1:400, 1:1200, and 1:1200, respectively. Collagen type‐I, α‐SMA, osteocalcin, and osteopontin staining were quantified as described for histomorphometry, using the same ROIs. The number of osteoblasts was counted manually in TRAP‐stained sections at the bone interface of ROItot. Blood vessels were classified and quantified as circular, intermediate, or irregular by α‐SMA in ROI400 μm.

### 2.5 Alveolar profile measurements

Individualized impression trays were fabricated for each animal. Silicon impressions of the mandible were taken using a light/heavy putty (Elite HD+, Zhermack spa, RO, Italy) prior to GBR procedure and at the end of study. Cast models were poured with stone (Fujirock type 4, GC. Corp, Tokyo, Japan), then optically scanned using a desktop 3D scanner (Zfx Evolution Scanner, Zimmer Dental, Bolzano, Italy) to obtain STL files. MeshLab 2022.02 was used to align the images.^16^ Buccolingual cross‐sections were made at the middle of the defect and exported to ImageJ for analysis. ROI was defined as the buccal half of the mandible, from the crest and 6 mm apically or until the mucogingival border. The impression taken prior to GBR procedure was set as a baseline and changes in area were measured in 2 mm increments in a coronoapical direction.^17^

### 2.6 Data analysis

Comparisons across groups were performed using parametric one‐way analysis of variance (ANOVA) for normalized datasets. Pairwise multiple comparison procedures were done by Holm–Sidak method. When the normality test or equal variance test failed, Kruskal–Wallis one‐way ANOVA on ranks was performed, and Dunn's method performed pairwise multiple comparison procedures. All statistical analyses were performed using SigmaPlot 14 (Systat Software, San Jose, California). Statistical significance was set at the 0.05 level.

## 3 RESULTS

### 3.1 Histology and histomorphometry

#### 3.1.1 Movat pentachrome

Mineralized bone was characterized by a dark yellow staining of the collagen. In intimate contact with this mineralized bone was a brighter yellow‐stained collagen network between the porous graft materials for both DBBM and TiO_2_ groups. This collagen network appeared homogenous but presented fibers in random orientation. Cell nuclei were evenly infiltrated within this collagen network. In addition, a collagen membrane covered the pristine bone in the negative control group and graft materials in DBBM and TiO_2_ groups.

**FIGURE 2:**
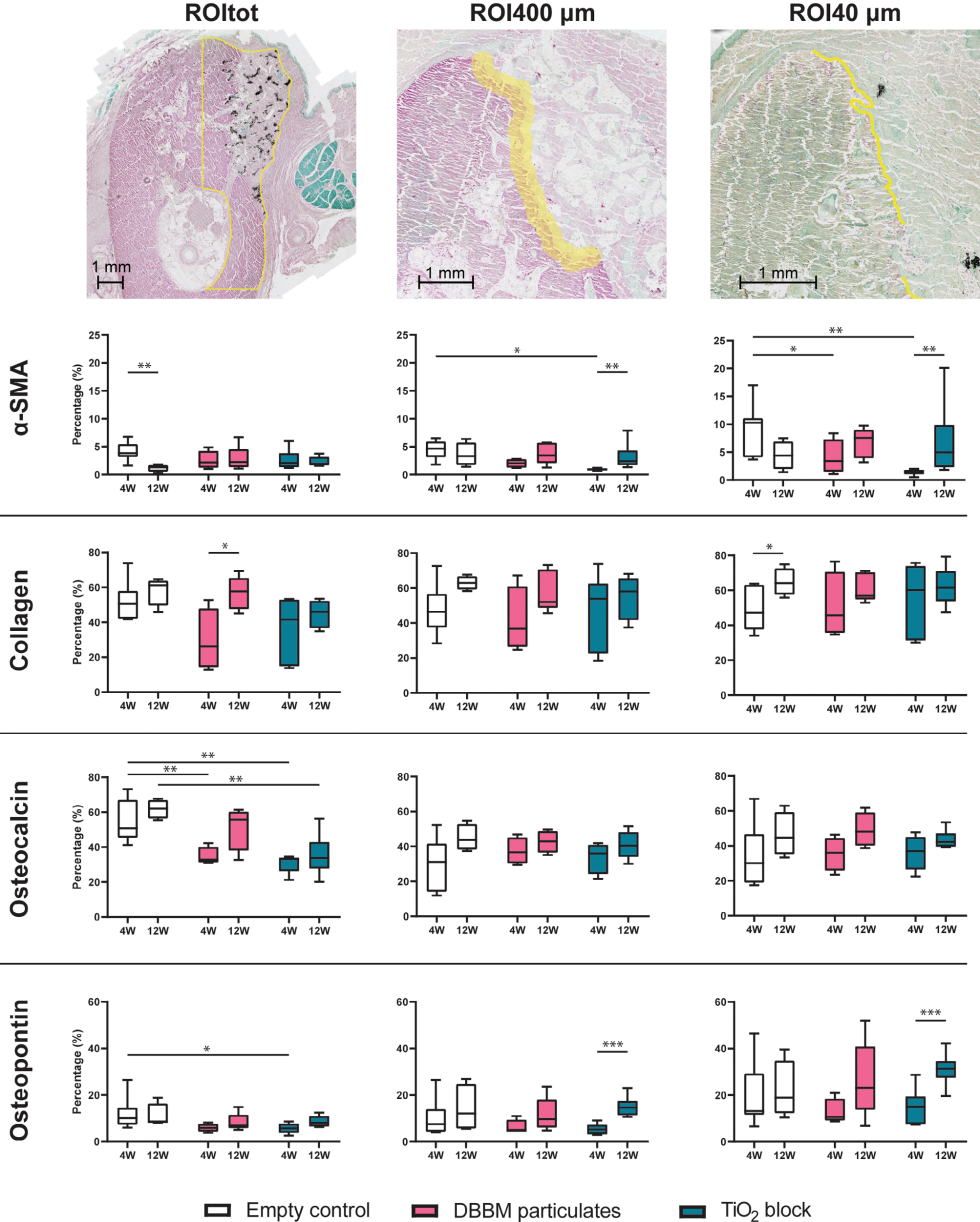
Illustrations of the different regions of interest (ROIs) analyzed. ROItot showing a TiO_2_ sample with osteocalcin stain, ROI400 μm showing a deproteinized bovine bone mineral (DBBM) sample with collagen stain and ROI40 μm showing a negative control sample with osteopontin stain. Area with graft material was excluded. Box plots with statistical significance denoted by asterisks: *p < 0.05, **p < 0.01, ***p < 0.001. α‐SMA, alpha‐smooth muscle actin

The fraction of collagen in the different ROIs is presented in (Figure 2). For ROI400 μm at 12 weeks healing time, a significant difference was found between empty control group (median: 87.56%, The interquartile range [IQR]: 81.21–90.46) and DBBM group (median: 68.68%, IQR: 64.72–76.51; *p* < 0.05). The collagen fraction decreased significantly from 4 weeks (median: 83.94%, IQR: 79.14–87.99) to 12 weeks for the DBBM group (*p* < 0.05). The same significant decrease was seen for DBBM group in ROI40 μm as well, from 4 weeks (median: 75.48%, IQR: 71.66–76.48) to 12 weeks (median: 67.97%, IQR: 42.34–70.90; *p* < 0.05).

#### 3.1.2 Von Kossa/Van Gieson

A loosely connected extracellular matrix was seen in contact with both DBBM and TiO_2_ graft materials and filled the space between the graft materials. The matrix was homogenous and similar for both DBBM and TiO_2_ groups.

**FIGURE 3:**
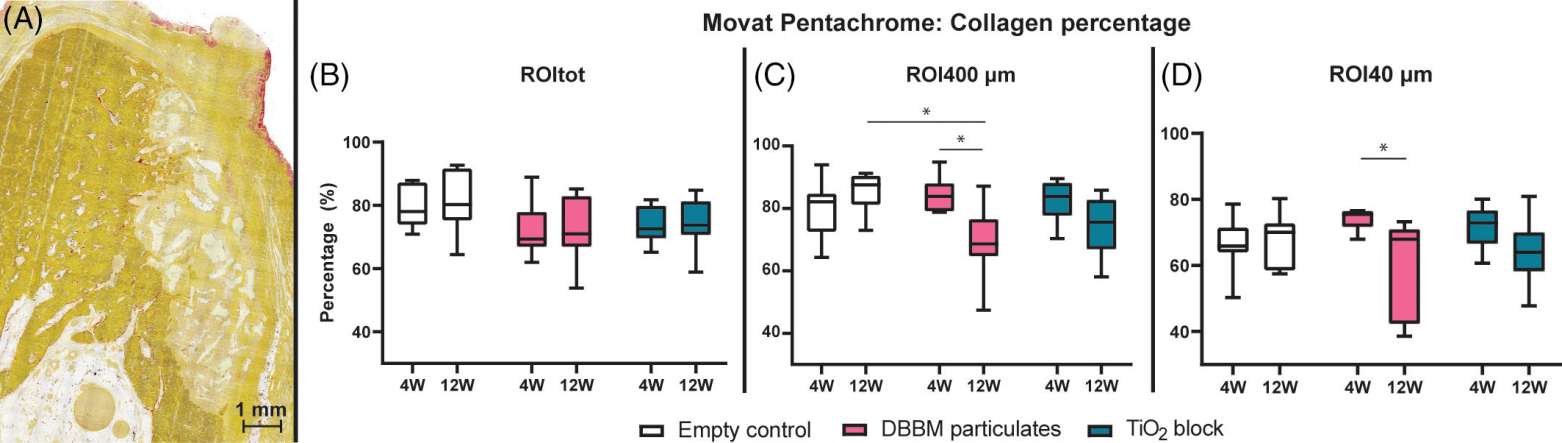
(A) Deproteinized bovine bone mineral (DBBM) particulates seen as light green, mineralized bone in dark yellow, and unmineralized collagen in bright yellow. Percentage of collagen in region of interest (ROI)tot (B), ROI400 μm (C) and ROI40 μm (D). Statistical significance denoted by asterisks: *p < 0.05. 4 W, 4 weeks; 12 W, 12 weeks

The fractions of the extra cellular matrix (ECM) are presented in Figure 3B–D. The empty control group exhibited a significantly lower fraction of ECM compared with DBBM group and TiO_2_ group at both 4 and 12 weeks. At 4 weeks: DBBM: median: 17.46%, IQR: 14.30–31.12, empty control: median: 6.50%, IQR: 4.03–8.30, TiO_2_: median: 16.97%, IQR: 16.06–25.74. At 12 weeks: DBBM: median: 14.68%, IQR: 9.51–18.44, empty control: median: 4.81%, IQR: 3.65–5.68, TiO_2_: median: 22.00%, IQR: 17.02–27.64.

### 3.2 Histochemical analyses

#### 3.2.1 Alpha‐smooth muscle actin

Blood vessels were evenly distributed in the mineralized bone with no apparent differences between the new and original bone of the alveolar ridge for both 4 and 12 weeks healing time points. The empty defects showed vascularization in the connective tissue buccal to the bone, but only small amounts were observed in the close vicinity of the cortical border (ROI400 μm and ROI40 μm). Both TiO_2_ group and DBBM group had ingrowth of blood vessels from the bone into the grafted area, but primarily at the border. Quantification of blood vessels by type at ROI400 μm; regular, moderately irregular, and irregular, demonstrated no differences between the group and timepoints (Table S1).

For the ROItot, the percentage of stained α‐SMA showed a significant decrease from 4 weeks (median: 3.83%, IQR: 3.16–5.47) to 12 weeks (median: 1.29%, IQR: 0.52–1.72) for empty control group (*p* ≤ 0.01). For ROI400 μm at 4 weeks, empty control (median: 4.68%, IQR: 3.21–5.95) was significantly higher than TiO_2_ group (median: 0.93%, IQR: 0.78–1.15; *p* < 0.05). The TiO_2_ group also showed a significant increase from 4 to 12 weeks (median: 2.41%, IQR: 1.70–4.33; *p* < 0.01). For ROI40 μm at 4 weeks, empty control (median: 10.26%, IQR: 4.07–11.10) was significantly higher than both DBBM group (median: 3.43%, IQR: 1.49–7.30) and TiO_2_ group (median: 1.47%, IQR: 1.03–1.81; *p* < 0.05). TiO_2_ group showed a significant increase from 4 to 12 weeks (median: 4.94%, IQR: 2.30–9.84; *p* < 0.01).

#### 3.2.2 Collagen

**FIGURE 4:**
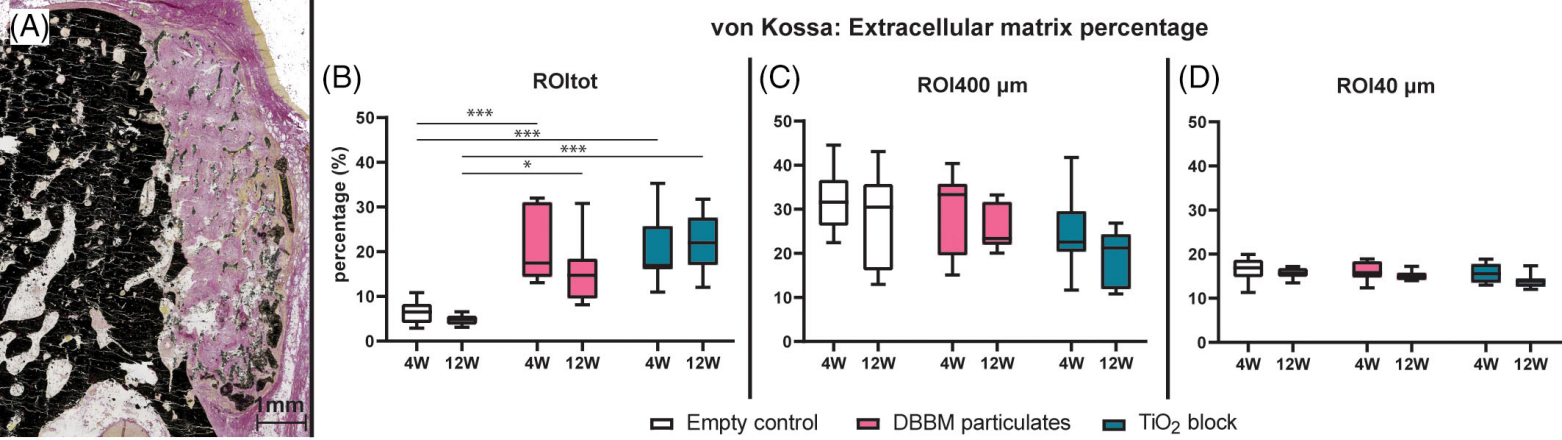
(A) TiO_2_ group. Mineralized bone, including deproteinized bovine bone mineral (DBBM) particulates, stained black by Von Kossa/Van Gieson, while TiO_2_ appeared dark gray to black. An extracellular matrix is stained pink within the TiO_2_ scaffold and presents less intensity than the surrounding soft tissue and epithelium. Some voids from fracture and tearing of the scaffold are seen. Note migrated DBBM particulates at the bottom of the defect. Percentage of extracellular matrix in region of interest (ROI)tot (B), ROI400 μm (C), and ROI40 μm (D). Statistical significance denoted by asterisks: *p < 0.05, ***p < 0.001

Collagen type I was detected by IHC in all samples. The mineralized bone was easily distinguishable with increased intensity along the bone border (Figure 4). For TiO_2_ and DBBM groups the intensity of collagen staining was weaker between the graft materials compared with mineralized bone, but similar to the surrounding extracellular matrix.

For ROItot a significant increase was seen for DBBM group from 4 weeks (median: 26.19%, IQR: 14.27–47.94) to 12 weeks (median: 57.76%, IQR: 47.54–65.35; *p* < 0.05). At ROI40 μm a significant increase was seen for empty control group from 4 weeks (median: 47.13%, IQR: 37.54–63.14) to 12 weeks (median: 64.20%, IQR: 57.58–72.60; *p* < 0.05).

#### 3.2.3 Osteocalcin

Osteocalcin intensity was similar across all groups and time points. For example, a homogenous staining of the prestine alveolar bone was observed, and no osteocalcin stain was seen in the grafted area.

For ROItot, at 4 weeks the negative controls (median: 50.83%, IQR: 45.34–67.03) were significantly higher than both DBBM group (median: 32.86%, IQR: 31.23–40.15) and TiO_2_ group (median: 33.17%, IQR: 26.30–33.98; *p* < 0.01). At 12 weeks, a significant difference was found between negative control (median: 62.01%, IQR: 56.35–66.98) and TiO_2_ group (median: 33.68%, IQR: 27.76–43.00; p < 0.01). No significant differences were found between the groups or from 4 to 12 weeks for ROI400 or ROI40 μm.

#### 3.2.4 Osteopontin

Osteopontin staining was found along the osteons' borders, resulting in a heterogeneous staining of the mineralized bone. Increased intensity was seen at the cortical borders of the defect site for all groups. Within the grafted areas, the intensity of osteopontin was weaker and observed on the surface of both DBBM and TiO_2_. Osteopontin was not seen in the space between the graft materials. For ROItot at 4 weeks, empty control (median: 10.09%, IQR: 4.28–7.64) was significantly higher than TiO_2_ group (median: 5.77, IQR: 3.71–7.71; *p* < 0.05). For ROI400 μm, TiO_2_ group demonstrated a significant increase from 4 weeks (median: 5.22%, IQR: 3.08–7.30) to 12 weeks (median: 14.66%, IQR: 11.31–17.45; *p* < 0.001). This was also seen at ROI40 μm for TiO_2_ group from 4 weeks (median: 15.08%, IQR: 7.41–19.49) to 12 weeks (median: 31.24%, IQR: 27.44–34.58; *p* < 0.01).

#### 3.2.5 Tartrate‐resistant acid phosphatase

**FIGURE 5:**
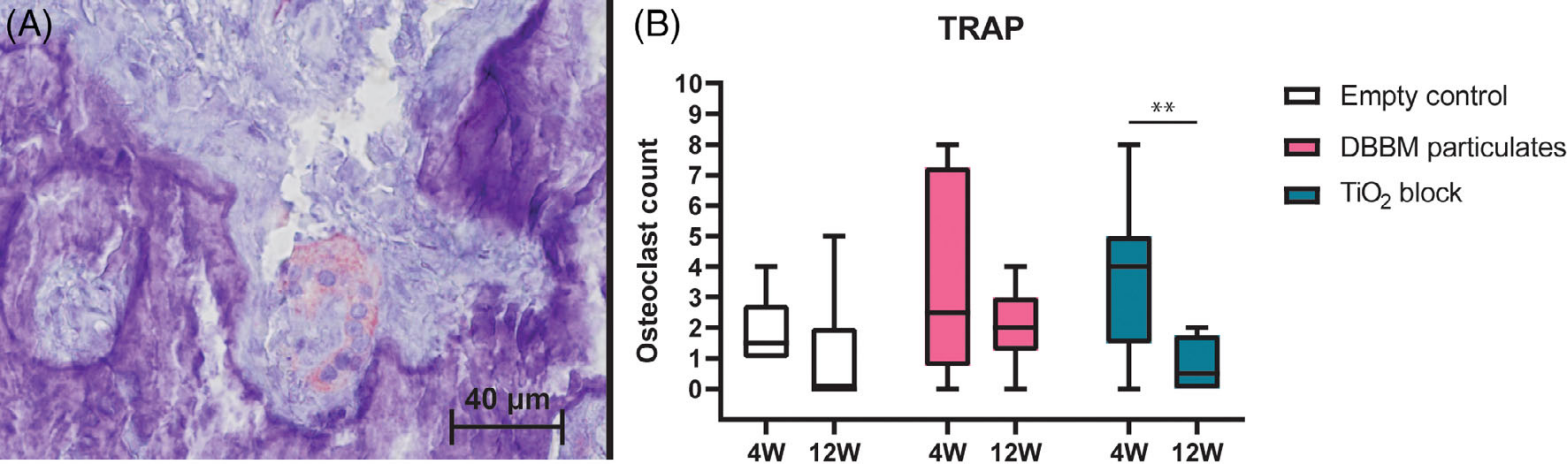
(A) Osteoclast in a Howship lacuna, (B) Double asterisks denote statistical significance (*p* < 0.01). DBBM, deproteinized bovine bone mineral; TRAP, tartrate‐resistant acid phosphatase

Osteoclasts were identified in 35 out of 46 samples within the ROI. Outside the ROI, osteoclasts were also found on the surface of the DBBM particulates close to the bone (Figure 5A). The TiO_2_ group demonstrated a significantly decrease in the number of osteoclasts from 4 weeks (median: 4.0, IQR: 1.5–5) to 12 weeks (median: 0.5, IQR: 0–1.75; *p* < 0.01; Figure 5B **)**.

### 3.3 Alveolar ridge profile

**FIGURE 6:**
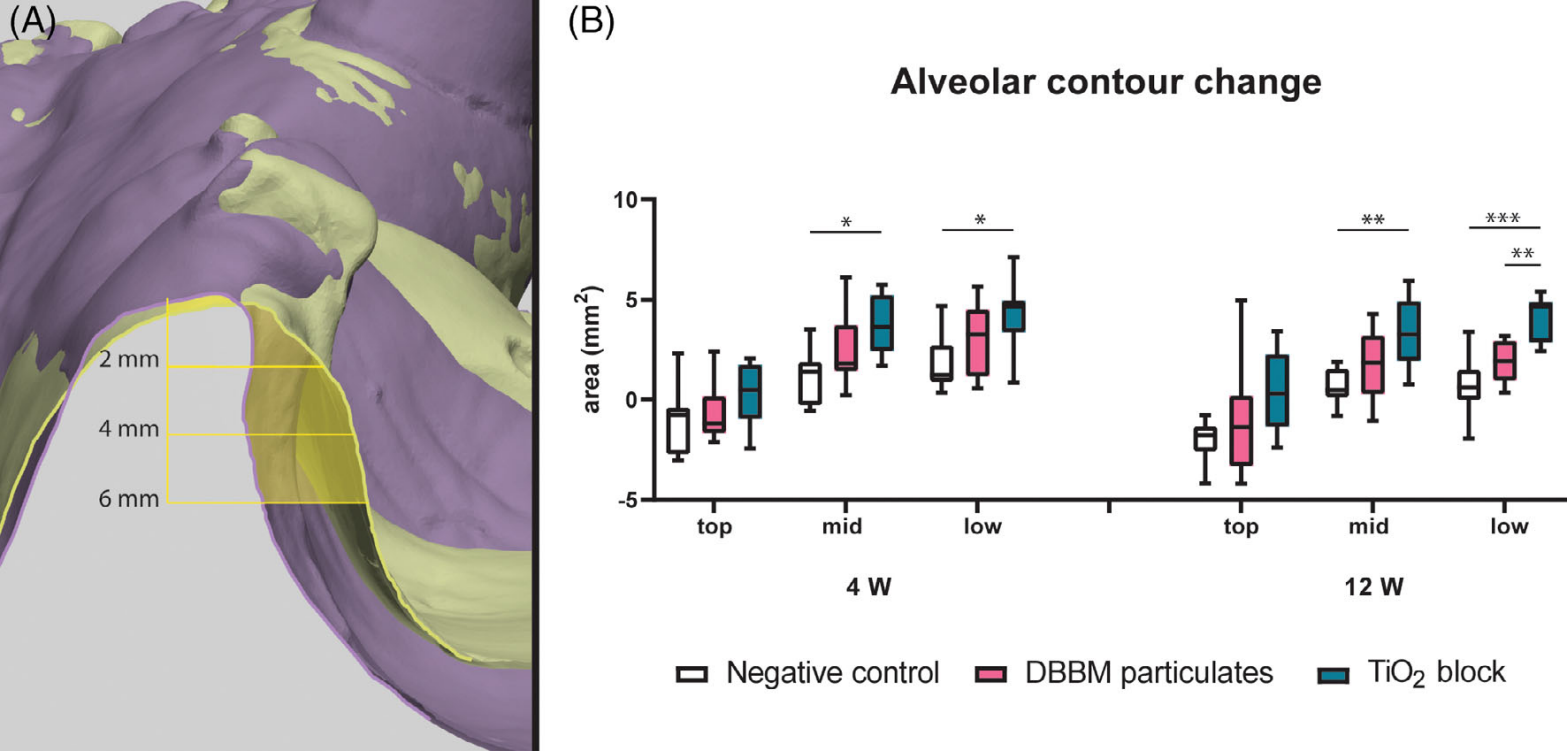
(A) Superimposed stereolithographic images of TiO_2_ sample 4 weeks after guided bone regeneration. The baseline in purple and 4 weeks healing in green. Area difference in ROI is illustrated in yellow. Shown distance from the alveolar crest divides the top, mid, and low segments. (B) Area differences for alveolar contour. Statistical significance denoted by asterisks: *p < 0.05, **p < 0.01, ***p < 0.001. DBBM, deproteinized bovine bone mineral

At 4 weeks of healing, a significant difference was seen between negative control and TiO_2_ group at the mid portion, median: 1.38 mm^2^, IQR: −0.30 to 1.88 and median: 3.62 mm^2^, IQR: 2.41–5.22, respectively (Figure 6B; *p* < 0.05). A significant difference between negative control and TiO_2_ group were also seen at the low portion, median: 1.24 mm^2^, IQR: 0.89–2.72 and median: 4.65 mm^2^, IQR: 3.38–4.96, respectively (*p* < 0.05). At 12 weeks healing, a significant difference was seen between negative control and TiO_2_ group at the mid portion, median: 0.47 mm^2^, IQR: 0.10–1.53 and median: 3.27 mm^2^, IQR: 1.92–4.92, respectively (*p* < 0.01). At the low portion, a significant difference between negative control (median: 0.61 mm^2^, IQR: −0.03 to 1.48) and TiO_2_ group (median: 4.66 mm^2^, IQR: 2.84–4.90; *p* < 0.001) and DBBM group (median: 1.93 mm^2^, IQR: 0.96–2.95; *p* < 0.01). No significant difference was observed from 4 to 12 weeks of healing in any group.

## 4 DISCUSSION

This study demonstrated that GBR in chronic defects using a TiO_2_ block led to an increase of α‐SMA and osteopontin from 4 to 12 weeks at the bone‐scaffold interface. In the DBBM group, an increase in collagen type I was observed for ROItot, while the negative control group showed decreased α‐SMA in ROItot and increased collagen type I at ROI40 μm. The findings suggest contact osteogenesis when GBR is performed with a TiO_2_ block or DBBM particulates. The increase in osteogenic markers indicates potential for bone formation beyond 12 weeks. In addition, the alveolar profile data showed a sustained lateral increase using a graft material against negative control.

These findings were in accordance with previous studies demonstrating osteoconductivity of TiO_2_ blocks in preclinical models.^7^, ^18^ In addition, osteoblasts' enhanced osteopontin and vascular endothelial growth factor secretion by osteoblasts on TiO_2_ have also been demonstrated in cell cultures compared with a SiO_2_ surface.^19^ The increased osteopontin at the bone border and around graft materials coincides with previous reports.^20^, ^21^, ^22^ Osteopontin is required for bone formation and facilitates osteoblast and osteoclast adhesion.^23^, ^24^, ^25^ As both osteopontin and blood vessels are essential for osteogenesis, an increase in osteopontin and α‐SMA from 4 to 12 weeks may indicate a potential for further bone growth. No uniform distribution of osteogenic markers could be found in any site, as can be done by in vitro assessment of osteogenic expression.^10^ However, this may be expected as a large site in vivo demonstrates different niches with varying osteogenic development in the augmented compartment. For a more homogeneous comparison, a ROI at the interface between bone and graft was considered to describe the osteogenesis across groups.

For the empty control group, α‐SMA‐stained blood vessels significantly decreased from 4 to 12 weeks in ROItot, but not in ROI40 and ROI400 μm. This change was not observed for DBBM or TiO_2_ groups. The different defect areas could explain this result. The regions evaluated for TiO_2_ blocks and DBBM particulates included the area with graft materials and the interface of bone and graft material, whereas empty control sites consisted of bone and adjacent soft tissue. This difference was evident by evaluating the extracellular matrix fraction with Von Kossa/Van Gieson stain, where ROItot constituted primarily mineralized bone in negative controls and no unmineralized grafted area. However, the results could also indicate a lower osteogenic activity at 12 than 4 weeks at negative control sites. Bone graft materials have been shown to delay initial bone regeneration as compared with empty control sites.^6^, ^26^ Faster initial healing at empty control sites covered by a collagen membrane was expected compared with DBBM‐grafted and TiO_2_‐grafted sites.

One advantage of using graft materials in GBR is space maintenance under the cell occlusive membrane.^27^, ^28^ It has been shown by microCT measurements that both DBBM particulates and TiO_2_ blocks preserved the space 12 weeks after GBR procedure. This study corroborated the results in alveolar contour change, which implied the soft tissue profile adapted to the graft materials. The STL images also made it possible to compare baseline alveolar shape prior to surgery with the shape after 4 and 12 weeks. A minor resorption was found at the top increment in the negative control group, as expected when a flap was raised and the bone crest exposed to perform GBR.^29^ A tissue loss at the coronal part was also found for the DBBM group but not for the TiO_2_ group. This may be attributed to the different handling and properties of materials used in the chronic, noncontained defects. For example, in the middle and lower part of the ridge, the TiO_2_ group was significantly wider than the empty control group. The DBBM group was not statistically significantly wider than the empty control group. This was expected, as a block graft material is more stable than a particulate. Graft dislocation following wound closure may also contribute to the reduced alveolar width at the coronal portion, especially for the particulates. As shown in an in vitro study by Mir‐Mari and colleagues,^30^ compressive forces on the augmented sites could not be totally avoided, even though a clinically tension‐free flap closure was achieved. The authors reported enhanced stability of the particulates by application of fixation pins or by the use of a block graft as compared with particulated bone substitutes. The effect of graft dislocation was found to be substantiated in one‐wall bone defects as compared with self‐contained defects.^31^ The authors found GBR with additional membrane fixation resulted in higher volume stability than without fixation and even better stability when a titanium‐reinforced membrane or a bone block was used. In this study, the collagen membrane was secured with metal pins. A reinforced membrane may have been beneficial for the particulate group; however, that would add another variable in the healing process. Additionally, DBBM with a resorbable collagen membrane is a commonly used and well‐documented procedure for augmentation and therefore chosen as a positive control.^32^

The results from the alveolar ridge profile measurements in this study were partly in agreement with reported findings from Di Raimondo and colleagues,^17^ who found a larger increase of the alveolar profile in the apical as compared with the coronal portions and a more significant increase for sites that included graft materials as compared with negative controls. However, the authors also demonstrated that a membrane alone significantly increased the alveolar contour, in contrast to this study. This may be explained by the difference in the defect model, as Di Raimondo and colleagues assessed peri‐implant dehiscence defects.

To the best of the authors' knowledge, few studies have reported the IHC characterization of lateral bone augmentation in chronic defects. Schwarz and colleagues^33^ reported on GBR with a biphasic hydroxyapatite and beta‐tricalcium phosphate compared with a collagen‐coated DBBM, whereas Cha and colleagues^34^ studied GBR using a biphasic calcium phosphate ceramic compared with DBBM and also empty defects without a membrane. Both authors reported on peri‐implant dehiscence defects in dogs, respectively, on acute and chronic defect models.

Cha and colleagues reported a significantly higher osteocalcin intensity at 8 weeks for sites treated with DBBM than sites treated with a biphasic calcium phosphate and empty sites with no membrane. At 16 weeks the osteocalcin intensity was similar across all groups. The authors observed osteocalcin around the mature bone. Schwarz and colleagues reported osteocalcin antigen reactivity in the connective tissue adjacent to DBBM and beta‐tricalcium phosphate granules. This was not seen in this study, where a stable intensity from 4 to 12 weeks was observed in mineralized bone only. The empty control group had the highest intensity in ROItot, but no differences were found otherwise. The lower intensity seen in the DBBM and TiO_2_ groups compared with this study's empty control group may indicate early osteogenesis as osteocalcin is a marker of the later stages.^35^

The comparable intensity of osteopontin was found across the groups at 8 and 16 weeks by Cha and colleagues. Osteopontin was situated around bone borders and graft particulates, according to this study. However, in this study, a higher intensity was found in the empty control group than the TiO_2_ group at 4 weeks for ROItot, and the TiO_2_ group demonstrated increased intensity from 4 to 12 weeks in both ROI40 and ROI400 μm.

Cha and colleagues reported no different TRAP counts for the DBBM group as compared with the empty control group, in agreement with this study. The biphasic calcium phosphate bone substitute demonstrated a significant increase in TRAP count from 8 to 16 weeks, which was hypothesized to be due to the resorption and following calcium and phosphate release from the biomaterial. As this study used a nonresorbable scaffold, no change in TRAP count was anticipated for the TiO_2_ group. In addition to the different models used by Schwarz and colleagues and Cha and colleagues, these studies also applied different methods for immunohistochemical analysis. Schwarz et al. used the cutting and grinding technique for MMA embedded samples,^36^ whereas Cha and colleagues embedded the samples in paraffin. The different techniques may further explain the different results found compared with this study.

When compared with a previous study,^7^ where methyl methacrylate sections were prepared by cutting and grinding, the use of microtome sectioned samples in this study presented several benefits. Above all, the 5 μm thin sections could be deplastified and decalcified after microtome sectioning. Decalcifying is usually performed on the bulk sample prior to paraffin embedding and sectioning. However, decalcification would not affect the ceramic TiO_2_ scaffold and would be impossible to cut when placed in soft decalcified tissue. The present method described by Malhan and colleagues^15^ allowed for histochemical staining of TiO_2_ containing samples without the need for specialized equipment like laser microtomes.^37^ By decalcifying 5 μm sections, the process was also quicker than bulk decalcifying. In addition, this technique yielded a higher number of sections as no material was lost by cutting and grinding. Ultimately, this may reduce the required number of animals, according to the principles of humane experimental technique.^38^ In this study, the thinner sections also resulted in better image quality. As previously reported, a dense structure was observed as a dark substance between the graft materials.^7^ With the thinner sections, a collagen network in the extracellular matrix was clearly identified from Movat Pentachrom and Von Kossa/Van Gieson stain. However, there were challenges with the methacrylate infiltration. Some samples were not adequately fixated. As a result, some sections had to be excluded in the analyses.

This study results should be interpreted with care due to the experimental nature of the study as well as the limited number of animals. In addition, the heterogeneity in study designs for GBR makes a comparison between studies challenging. The low number of treatment groups was also a limitation, and a DBBM material in block configuration could have served as a more relevant control in this study design. Further studies should also evaluate if the regenerated tissue obtained with TiO_2_ blocks will be stable over time and allow a reliable osseointegration of implants. Finally, despite the indications of osteogenic differentiation, a longer observation time is required to confirm future bone formation.

## 5 CONCLUSION

In conclusion, within the study's limitations, the findings suggest contact osteogenesis when GBR is performed with a TiO_2_ block or DBBM particulates. The increase in osteopontin markers indicates potential for osteogenesis beyond 12 weeks in this model. However, the alveolar profile data indicated a sustained lateral increase in lateral bone augmentation using a TiO_2_ block and a collagen membrane, as compared with DBBM and a collagen membrane or a collagen membrane alone.

## AUTHOR CONTRIBUTIONS

Mariano Sanz and Håvard Jostein Haugen prepared the protocol, acquired funding, and critically revised the article. Minh Khai Le Thieu, Sabine Stoetzel, Maryam Rahmati, Thaqif El Khassawna, Anders Verket, Javier Sanz‐Esporrin, Jan Eirik Ellingsen, and Håvard Jostein Haugen conducted the study. Minh Khai Le Thieu and Anders Verket interpreted data/analysis. Anders Verket and Javier Sanz‐Esporrin led the study. All authors contributed in critical revision of the article. The data that support the findings of this study are available from the corresponding author upon reasonable request.

## CONFLICT OF INTEREST

Haugen and Ellingsen hold patents for the technology for the TiO_2_ bone graft substitute (EP Patent 2121053, US Patent 9629941 US Patent App. 14/427901, US Patent App. 14/427683, and US Patent App. 14/427854). The rights for these patents are shared between the University of Oslo and Corticalis AS. Haugen and Ellingsen are shareholders and board members of Corticalis AS. The other authors report no conflicts of interest related to this study.

## Supporting information


**TABLE S1** Classification of blood vessels (median)

## Data Availability

The data that support the findings of this study are available from the corresponding author upon reasonable request.
