# Supplementary material for: Immunohistochemical comparison of lateral bone augmentation using a synthetic TiO2 block or a xenogeneic graft in chronic alveolar defects
Source: Clin Implant Dent Relat Res. 2022 Oct 12;25(1):57–67. doi: 10.1111/cid.13143 (PMC10092822; doi:10.1111/cid.13143)
Supplement: Supplementary file 1 — TABLE S1 Classification of blood vessels (median) [file CID-25-57-s001.docx]

| Classification of blood vessels (median) | | |
| --- | --- | --- |
|  | **4W** | **12W** |
| **DBBM** |  |  |
| Regular | 0 | 0 |
| Moderately irregular | 1 | 0 |
| Irregular | 0 | 2 |
|  |  |  |
|  |  |  |
| **Empty control** |  |  |
| Regular | 0 | 0 |
| Moderately irregular | 0 | 0.5 |
| Irregular | 0 | 1 |
|  |  |  |
|  |  |  |
| **TiO2** |  |  |
| Regular | 0.5 | 0 |
| Moderately irregular | 0.5 | 0.5 |
| Irregular | 0 | 2 |
